# Survival in Patients with Primary Parotid Gland Carcinoma after Surgery—Results of a Single-Centre Study

**DOI:** 10.3390/curroncol30030204

**Published:** 2023-02-23

**Authors:** Filippo Carta, Mauro Bontempi, Daniele De Seta, Simone Corrias, Melania Tatti, Valeria Marrosu, Cinzia Mariani, Clara Gerosa, Sanjana Ashik Shetty, Matteo Atzeni, Christina Buckley, Andrea Figus, Roberto Puxeddu

**Affiliations:** 1 Otorhinolaryngology Unit, Department of Surgery, Azienda Ospedaliero-Universitaria di Cagliari, University of Cagliari, 09124 Cagliari, Italy; 2 Pathology Unit, Department of Surgery, Azienda Ospedaliero-Universitaria di Cagliari, University of Cagliari, 09124 Cagliari, Italy; 3 Guy’s and St Thomas’ NHS Foundation Trust, London SE1 9RT, UK; 4 Plastic and Reconstructive Surgery Unit, Department of Surgery, Azienda Ospedaliero-Universitaria di Cagliari, University of Cagliari, 09124 Cagliari, Italy

**Keywords:** parotid cancer, parotid tumour, primitive parotid malignancy, salivary gland tumour, parotidectomy

## Abstract

This study aims to analyse a single-centre cohort series of patients who underwent parotidectomy for primary malignant parotid tumours. A retrospective chart review of 64 consecutive patients treated from November 2010 to March 2022 was performed. Outcomes were analysed by Kaplan-Meier curves. Sixty-four patients with a primary parotid malignancy were included in the study, with one bilateral case in this cohort. Patients were classified as stage I–II in 39 cases and stage III–IV in 26 cases. The five-year overall survival (OS), disease-specific survival (DSS), local relapse-free survival (LRFS), and distant metastasis-free survival (DMFS) rates were 78.4%, 89%, 92.5%, and 87.1%, respectively. Univariate analysis showed that high-risk histology, stage IV disease, lymphovascular invasion, perineural invasion, node metastasis, skin involvement, facial nerve involvement, and positive or close margins were risk factors associated with poorer outcomes. At present, the best evidence suggests that radical surgery should be the standard approach, and adjuvant therapy, in terms of radiotherapy/chemoradiotherapy, is recommended in patients with risk factors.

## 1. Introduction

Salivary gland tumours represent a heterogeneous group of neoplasms that widely vary according to histological type and grade [1]. These tumours had an overall worldwide incidence of 0.57 cases per 100,000 in 2020 [2], and malignant histotypes represent 1–6% of all head and neck carcinomas and 0.3% of all cancers [3].

Males are more frequently involved than females, with a sex ratio M/F of 1.7:1 [4].

The higher incidence of cases is found in patients aged between 60 and 70 years [5]. Tumours in younger patients are rare and usually associated with a higher frequency of malignant histotypes [5].

There are no shared theories about the aetiology of these tumours, even if different hypotheses have been proposed, such as gamma radiation exposure [6] and occupational exposure to nickel compounds and rubber [7].

Approximately 60% to 70% of all salivary gland tumours originate in the parotid gland, of these about 20% are malignant [8].

The fourth edition of Histology Classification published in 2017 by the World Health Organisation characterises 20 malignant histotypes [9]. This heterogeneity makes the management of parotid cancers really challenging [10].

Clinical presentation of early stage malignant lesions is not different from benign or non-neoplastic lesions [11], and the differential diagnosis is complex: 94% of the benign lesions and 66% of malignant lesions have only a parotid swelling without any other sign or symptom [12,13].

Radiological imaging and cytopathology diagnosis attempt to differentiate between early malignancies and benign lesions, setting different clinical priorities in case of suspected cancer.

The main treatment modality for parotid malignancies is surgery. Neck dissection and postoperative radiotherapy (PORT) are indicated for patients with adverse pathologic features, such as T3–T4 lesions, neck metastasis, perineural invasion (PNI), lymph vascular (LVI) invasion, and high-risk histotypes [1,10,13].

The primary aim of the current study is to report our experience in managing patients diagnosed with a primary parotid gland malignancy who underwent surgery, while the secondary aim was to analyse the prognostic factors affecting outcomes.

## 2. Materials and Methods

A retrospective analysis approved by the Ethical Committee on Human Clinical Research of our Hospital (PG/2016/4730) was conducted on 64 consecutive patients with primary parotid gland malignancy who underwent surgery from November 2010 to March 2022.

Ultrasonography (US), magnetic resonance imaging (MRI), and computed tomography (CT) were used for preoperative evaluation.

Fine needle aspiration cytology (FNAC) has been routinely performed in our department since 2016 and cytological diagnosis was reported according to the Milan System for salivary gland [14].

All patients were staged according to the 8th edition of the TNM staging system [15].

Lesions were classified as low-risk and high-risk according to the WHO Classification of Head and Neck Tumours [9].

Parotidectomies were classified according to Quer [16]. We never performed enucleation or extracapsular dissection of the tumour.

Patients with lesions involving the surrounding skin underwent en-bloc resection and primary reconstruction with free or pedicled flaps.

Neck dissection (concomitant or delayed) and adjuvant therapies were performed according to the NCCN Guidelines for Head and Neck Cancers [17].

Facial nerve (FN) function was evaluated preoperatively and postoperatively at Day 1, 1 month and at least 6 months after surgery using the House–Brackmann grading system scale (HB) [18]. One patient with preoperative grade III facial palsy because of a previous parotidectomy performed in another institution was excluded from the analysis of FN outcomes. FN dissection was always performed under microscopic view. Systematic intraoperative FN monitoring was always performed using a four-channel free-running electromyography nerve integrity monitor system (NIM 3.0, Medtronic USA, Inc., Jacksonville, FL, USA).

Corticosteroids (dexamethasone, 4 mg/day for 7 days) were used when immediate postoperative FN weakness was present.

The following risk factors were analysed: sex; preoperative facial pain; age at diagnosis; histological grade of malignancy (low risk versus high risk); tumour size, stage, and nodal involvement; skin invasion; FN involvement; PNI; LVI; positive or close surgical margins.

The definition of close margins in cancers originating from major salivary glands is not well defined in the literature. We considered a free resection margin < 5 mm as a close surgical margin, as suggested by other authors [19,20].

Statistical analysis was performed using GraphPad Prism 9 (San Diego, CA, USA), and a *p*-value < 0.05 was considered significant. Kaplan–Meier curves were calculated for survival outcomes. Disease-specific survival (DSS), overall survival (OS), local relapse-free survival (LRFS), and distant metastasis-free survival (DMFS) were considered endpoints. The log-rank and Wilcoxon tests were used to compare the survival curves. The chi-square test was used for categorical variables. The Bonferroni method was used to evaluate the reliability of multiple comparisons (stage of disease, margin status and tumour’s size) in order to control the family wise error rate. Lastly, a multivariate analysis was performed (Supplementary File S1), but it did not show any statistical significance, probably because of the low number of positive results of the dependent variable (dead of disease).

## 3. Results

Sixty-four consecutive patients (21 M, 43 F, ratio M/F 0.5) with primary parotid gland carcinoma were definitively included in the study (Table 1 and Supplementary File S2). In one case, the tumour was bilateral.

Seven patients had a clinical history of previous parotid surgery performed elsewhere: two patients were treated for pleomorphic adenoma; one patient was previously treated for acinic cell carcinoma 20 years prior; one patient was treated with multiple excision of myoepithelial carcinoma 8 years prior; one patient for recurrent low-grade adenocarcinoma treated 2 years prior; one patient for recurrent cystic adenocarcinoma treated 3 years prior; one patient for recurrent acinic cell carcinoma treated 3 years prior.

Clinical features included parotid mass without any other symptoms in 43 cases (67.2%) and a painful parotid lump in 17 cases (26.7%) associated with overlying skin involvement in 3 cases. Preoperative FN palsy was observed in three cases (4.7%).

Four cases (6.1%) were incidentally diagnosed during an MRI follow-up for other reasons.

Preoperative work-up was performed with US and MRI in 39 cases, MRI alone in 13 cases, CT scan with both MRI and US in 4 cases, both CT and MRI in 9 cases, CT alone in 4 cases, and both CT scan and US in 3 cases. Preoperative diagnosis of malignancy was obtained by incisional biopsy or Tru-Cut in seven patients with locally advanced lesions. Twenty-eight patients underwent FNAC (diagnosis of malignancy in fourteen cases and not diagnostic or suggestive for benign lesions in fourteen cases). The overall sensitivity of FNAC was 50%. The sensitivity of FNAC performed without US guidance before 2016 (n = 7) was 28.6%, while the sensitivity of FNAC performed with the aid of US (n = 21) was 57.1%.

The surgical procedures performed are shown in Table 1.

In three patients, parotidectomy included resection of the overlying skin and free flap reconstruction was performed (radial forearm n = 1, anterolateral tight flap n = 1, and rectus abdominis n = 1).

Concomitant neck dissection was performed in all 13 cN+ patients.

Indication for delayed neck dissection was made in a multidisciplinary team discussion for 10 cN0 patients after a precise histological assessment. Three of them accepted the procedure while seven refused neck dissection and underwent prophylactic radiotherapy (RT) of the neck.

Forty-one cN0 patients (patient with bilateral lesions was cN0 bilateral) underwent follow-up without indication for any other form of treatment.

In 48 out of 64 (75%) procedures, the FN was preserved, and the patients did not experience any long-term postoperative palsy. Five patients (7.8%) experienced grade II permanent palsy of the marginal mandibular branch despite its intraoperative anatomic preservation. Nine patients (14.1%) without preoperative facial weakness experienced postoperative permanent palsy limited to a single branch of the nerve that was resected for macroscopic involvement observed intraoperatively and demonstrated with frozen sections (nerve grafting with the great auricular nerve was performed in 5 cases during the same procedure). Two patients (3.1%) with FN involvement diagnosed before surgery underwent complete resection of the nerve in one case (nerve grafting using the great auricular nerve to the distal branch of the orbicularis nerve, static facial rehabilitation, and tarsorrhaphy were performed during the same procedure) and resection of the temporo-facial branch in the other case (nerve graft using the great auricular nerve was performed).

We observed 24 postoperative complications (Table 2).

Histological diagnoses are reported in Table 1. Forty-six lesions (70.8%) were classified as low risk according to the risk stratification of the WHO and nineteen (29.2%) were classified as high risk by histology and grade [21].

Thirty patients (46.9%) underwent PORT (four patients with stage I, six patients with stage II, eight patients with stage III, and twelve patients with stage IV), one patient did not undergo adjuvant therapy despite an advanced stage of disease, as it was contraindicated due to scleroderma, and in two further cases of high-grade malignancy, RT could not be performed due to previous neck irradiation (previous supraglottic SCC in one case and recurrent parotid adenocarcinoma in the other case; the former case developed recurrence of the tumour in the neck and was treated with therapeutic neck dissection).

Two patients (3.1%) with stage IV disease, positive margins, and associated neck metastases, with extranodal extension (ENE) in one case, underwent concurrent chemoradiotherapy (CRT).

All patients underwent follow-up (mean time 3.57 years, median time 3.61 years; range, 6 months to 10 years).

During the follow-up, one patient (1.6%) experienced recurrence of oncocytic carcinoma within the parapharyngeal space (previous treatment was type I-II-III parotidectomy) and underwent neck dissection and PORT (in this case, the transcervical approach allowed the FN to be spared, and the patient was alive without any signs of persistence/relapse of disease at the last follow-up visit). Two patients (3.1%) experienced recurrence of the disease in the neck after 1.6 and 0.8 years after primary surgery and underwent neck dissection (the patients were alive without evidence of disease at the last follow-up visit). Seven patients (10.9%) experienced single distant metastasis; all these patients underwent chemotherapy, five of them died from the disease while two are still alive.

The overall incidence of neck metastasis was 16.9% (11/65): neck metastasis was confirmed in eight of the thirteen cN+ patients (61.5%) treated with concomitant neck dissection, and occult metastasis was observed in three out of fifty-two cases (5.8%) in the cN0 group (one occult metastasis was observed after delayed neck dissection, and two neck metastases were detected during the follow-up). High-risk histology (31.6% versus 10.9%, *p* < 0.05) and T3℃T4 stages in low-risk histotypes (33% versus 2.9%, *p* < 0.05) were associated with a higher incidence of neck metastasis (Table 3).

The five-year OS, DSS, LRFS, and DMFS rates were 78.4%, 89%, 92.5%, and 87.1%, respectively (Table 4).

Univariate analysis showed that high-risk histology, stage IV disease, LVI, PNI, node metastasis, skin involvement, FN involvement, and positive or closed margins were risk factors associated with poorer outcomes (Table 5). Sex, preoperative facial pain, age at diagnosis (≥55 years or ≥65 years), tumour size, and ENE were not associated with worse outcomes (Table 5).

## 4. Discussion

The preoperative differential diagnosis between benign and malignant salivary gland tumours can be challenging because low-grade malignant parotid tumours generally present initially as slow-growing masses, displacing instead of infiltrating surrounding structures. Previous studies have reported that only one-third of malignant lesions present initially with facial palsy, skin infiltration, or obvious neck metastasis as clinical signs of malignancy [13]. In our series, 67.2% of patients with primary parotid malignancy presented with parotid swelling only and had no other accompanying red flag symptoms.

The most common imaging techniques used in the diagnosis of parotid tumours are CT scans and MRI. Imaging can reveal the features of the tumours, the anatomy of the adjacent structures, and obvious nodal metastases [22].

FNAC has become a standard diagnostic test of parotid masses, with an overall diagnostic accuracy of 96% (95% CI, 94–97%) in distinguishing benign from malignant parotid tumours [13]. Our results showed a sensibility increment of FNAC when US-guided, as observed by other authors [13,23].

Surgery remains the gold standard, followed by PORT in the presence of risk factors [24]. Surgical treatment of patients with a parotid tumour should be the radical resection of the lesion without any neoplasm seeding, ideally preserving the entire FN integrity. In our series, the parotidectomy was extended to the deep lobe when the tumour was confined in it (five cases) or if there was evidence of direct spread (nine cases). A clear histological resection margin was obtained in 86.2% of patients. In nine cases (13.8%), the resection margins were clean, but the tumour was closer than 5 mm from the margins (R close). Patients with extensive tumours that could not be treated by radical resection with a negative margin of more than 5 mm had a worse prognosis. As a consequence, complete resection of the parotid gland and removal of the surrounding areas are indicated in patients with locally advanced disease [25,26].

FN dissection can be performed using the loupes, the operative microscope and, more recently, the 3D exoscopic technology [27]. According to the literature, the rate of neoplastic involvement of the FN is 12–40% (16.9% in our series of patients) [28]. In the case of normal preoperative FN function, when the nerve is not involved by the lesion, all efforts should be made to preserve its integrity during surgery [25,28]. In the case of uncertain nerve involvement (when there is normal FN function preoperatively), a frozen section is needed to confirm the neoplastic involvement (intraoperative diagnosis of nerve infiltration was found in nine cases in our cohort) [25]. In the case of preoperative palsy for macroscopic infiltration of the FN, radical parotidectomy is the standard approach, and the branches of the nerve encased by the tumour should be surgically resected [13,25,28]. In our series, the nerve was resected when macroscopically involved, or in case of histological evidence of nerve infiltration at frozen sections. Facial reanimation was performed using immediate autografting (using the greater auricular nerve as the donor nerve graft) in seven cases. Tarsorrhaphy was also performed in two patients due to extensive resection of the nerve. Nerve grafting was associated with an improvement in facial muscle tone in all patients within three months after surgery. In our analysis, FN involvement was associated with a worse prognosis (higher incidence of distant metastasis) despite radical resection, according to Park et al. [29].

In the literature, the most frequently involved neck levels in patients with primary salivary gland malignancies are II, III, and IV [30]; levels I and V can also be involved, especially in high-grade tumours [13]. In our series, neck node metastases were observed in 11 patients: 8 (72.7%) in level II; 1 in (9.1%) level I; 2 in (18.2%) multiple levels (II, III, IV, and V). While neck dissection and adjuvant therapy in N+ patients are generally recommended, the management of cN0 has more treatment options. In such cases, the possible presence of occult metastases is the main problem. In our series, the occult metastasis rate in cN0 patients was 5.8% (in the literature, the incidence of occult metastases is 12–49%) [31]. Kaura et al. supports the use of routine neck dissection only for the treatment of high-grade lesions, invasive carcinoma ex pleomorphic adenoma, salivary duct carcinoma, high-grade adenocarcinoma not otherwise specified, adenoid cystic carcinoma, and high-grade acinic cell carcinoma [32]. In selected cN0 patients with T1/T2 tumours and low-grade histology with free surgical margins, observation may be applied [33]. In our series, patients with T1–T2 low-risk lesions experienced a lower incidence of neck metastasis of 2.9%, while patients with T3–T4 low-risk lesions and patients with T1–T2 and T3–T4 high-risk lesions experienced neck metastasis rates of 33%, 44.4%, and 20%, respectively (Table 3). Lombardi et al. suggested performing a super-selective (level I–II) or selective (I–III) neck dissection with frozen sections in cases of high-risk occult nodal disease that could be extended to a comprehensive neck dissection if the nodal disease is confirmed intraoperatively [31]. Elective neck irradiation (ENI) instead of delayed neck dissection in the case of risk factors for occult disease diagnosed at the definitive histology (high-grade and/or T3–T4 tumours) is a therapeutic option that, in our opinion, should be considered instead of neck dissection, mostly in patients already suitable for PORT for the primary tumour [31]. ENE was observed in five cases (7.7%) and was not clearly associated with a worse outcome (Table 5): one patient experienced distant metastasis within the first year of follow-up; two patients died due to other causes after 4 and 3.6 years of follow-up; the other two patients were alive but with distant metastatic disease after 2.8 and 1.3 years of follow-up.

The univariate analysis showed that lesions macroscopically involving the skin have a higher incidence of distant metastasis; in such cases, patients in our series underwent extended resections followed by adjuvant therapy. Significant defects of the parotid area can be disfiguring and profoundly impact quality of life, especially when associated with FN resection. Plastic surgery and FN rehabilitation should be planned as a single-stage procedure, and large defects are best replaced with vascularized tissue to provide adequate volume. In our series, three patients required reconstruction of the parotid area with a microvascular free flap. Following PORT, we had no major complications in our patient cohort. Minor complications included skin erythema, pain, and oral mucositis.

According to the literature, PORT is indicated for advanced-stage disease (T3/T4), intermediate- or high-grade tumours, close or positive margins, lymph-node metastases, bone invasion, PNI, and LVI [13]. In our series, 4 of the 17 patients with stage I disease underwent PORT (23.5%), 6 of the 22 patients with stage II disease underwent PORT (27.3%), 8 of the 10 patients with stage III disease underwent PORT (80%), 12 of the 16 patients with stage IV disease underwent PORT (75%), and 2 patients (12.5%) underwent CRT.

In the present study, DSS and DMFS were found to be related to high-risk histology, tumour stage, LVI, PNI, skin involvement, FN involvement, and positive or close resection margins and neck metastases.

Local recurrence was associated with high-risk histology, tumour stage, and nodal involvement; patients who experienced isolated local recurrence were managed successfully with salvage surgery and radiotherapy.

Sex, preoperative facial pain, tumour size, and ENE were not predictors of postoperative outcomes.

Molteni et al. observed that an age at diagnosis greater than 55 years was a cause of poorer disease survival [10]. In our cohort of patients, comorbidities (and, therefore, performance status), histological types, and oncological stages were more homogeneously distributed in both groups; as a consequence, age alone was not associated with a poorer prognosis.

In our series, distant metastasis was the main cause of disease-specific death.

A possible limitation of our study is represented by the low number of patients dead of disease: therefore, our multivariate analysis does not reach high statistical significance and the present results are supported only by the univariate analysis.

First-line RT and CRT are usually indicated for patients with unresectable tumours or those who decline surgery.

Recently, Hadron therapy, with the use of protons and carbon ions, has been reported as an effective treatment option in cases of inoperable patients or with locally advanced parotid gland carcinomas or in cases of recurrence requiring reirradiation [34,35]. According to Koto et al., patients should be counselled about the options for alternative nonsurgical treatments for cases of advanced disease that would require radical resection resulting in FN sacrifice [34].

The prognosis in case of inoperable, recurrent and metastatic disease remains poor. In such cases, different systemic therapeutic options are available. Recently, targeted therapies (e.g., antibodies against c-Kit, EGFR, VEGFR, ErbB2/HER-2) have been introduced when tumour-specific targets are available. However, no randomized controlled trials compare survival outcomes between different targeted systemic therapies in patients with salivary gland carcinomas. Chemotherapy can be always used, while few clinical trials have been conducted regarding the efficacy of immunotherapy targeting PD-1/PD-L1 and CTLA-4 [36].

## 5. Conclusions

In our series, the risk factors mainly associated with poorer outcomes were high-risk histology, stage IV disease, LVI, PNI, node metastasis, skin involvement, FN involvement, and positive or close margins. Clinical stage and FN involvement were greater predictors of the extent of surgery required than histological type and grade. Radical surgery is the standard of care, while adjuvant therapy in terms of radiotherapy or chemoradiotherapy is recommended in patients with risk factors. Proton beam therapy could be useful to improve outcomes in patients with stage IV disease, in which prognosis remains poor despite the use of aggressive surgical and adjuvant therapies. Further studies are required to better investigate the reliability of these alternative treatments.

## Figures and Tables

**Table 1:** Demographics and clinical data.

Patients		n (%)
Age (median range)	60.5 years to 4–83 years	64 *
Age (at the time of first surgery)	<55	27 (42.2%)
	≥55	37 (57.8%)
Sex	Male	21 (32.8%)
	Female	43 (67.2%)
	Ratio (M/F)	0.48
Side of tumour	Right	32 (50%)
	Left	31 (48.4%)
	Bilateral	1 (1.6%)
Lobe	Superficial	50 (78.1%)
	Deep	6 (9.4%)
	Both	9 (12.5%)
Preoperative work-up	US	42 (65.6%)
	CT	16 (25%)
	MRI	57 (89%)
	FNAC	28 (43.7%)
	Biopsy	7 (10.9%)
Surgical treatment	Parotidectomy I	0 (0%)
(according to Quer’s classification)	Parotidectomy I-II	32 (49.2%)
	Parotidectomy I-II-III	14 (21.5%)
	Parotidectomy I-IV	9 (13.8%)
	Parotidectomy I-IV (VII)	10 (15.5%)
Reconstructive surgery	Radial forearm free flap	1 (1.5%)
	Rectus abdominis free flap	1 (1.5%)
	Anterolateral thigh free flap	1 (1.5%)
	No	62 (95.4%)
Clinical nerve involvement (CNI)	Yes	3 (4.6%)
	No	54 (83%)
Facial nerve resection	Partial	10 (15.5%)
	Complete	1 (1.5%)
House–Brackmann (>6 month)	I	48 (75%)
	II	8 (12.5%)
	III	5 (7.8%)
	IV	2 (3.1%)
	V	1 (1.6%)
	VI	0 (0%)
Neck dissection	No	46 (70.8%)
	Yes	19 (29.2%)
	Concomitant	13
	Delayed	3
	Neck recurrence	3
Tumour’s size	≤2 cm	23 (35.4%)
	>2–≤4 cm	37 (56.9%)
	>4 cm	5 (7.7%)
Histological diagnosis	Acinic cell carcinoma	16 (24.6%)
	Mucoepidermoid carcinoma	13 (20%)
	Carcinoma ex pleomorphic adenoma	8 (12.3%)
	Adenoid cystic carcinoma	8 (12.3%)
	Myoepithelial carcinoma	4 (6.2%)
	Secretory carcinoma	3 (4.5%)
	Primitive squamous cell carcinoma	3 (4.5%)
	Oncocytic carcinoma	3 (4.5%)
	Adenocarcinoma	2 (3%)
	Salivary duct carcinoma	2 (3%)
	High grade carcinoma	2 (3%)
	Undifferentiated carcinoma	1 (1.5%)
pT stage	pT1	17 (26.1%)
	pT2	26 (40.0%)
	pT3	13 (20.0%)
	pT4a + pT4b	9 (13.9%)
pN stage	pN0	54 (83.1%)
	pN+	11 (16.9%)
	ENE+	5/11 (45.4%)
Stage	I	17 (26.1%)
	II	22 (33.8%)
	III	10 (15.4%)
	IVa + IVb	16 (24.7%)
Status of margins	R0	43 (66.2%)
	R close (<5 mm)	13 (20%)
	R1	9 (13.8%)
Adjuvant treatment	Radiotherapy	30 (46.9%)
	Chemoradiotherapy	2 (3.1%)
	No	32 (50%)
Relapse	No	53 (81.5%)
	N recurrence	3 (3%)
	Distant metastasis	7 (10.8%)
Persistence	T	1 (1.5%)

* Sixty-four patients, 65 lesions.

**Table 2:** Postoperative complications.

Patients n (%)	Complication	Treatment
16 (25.6%)	Permanent earlobe numbness	None
6 (9.6%)	Frey’s syndrome	None
1 (1.6%)	Bleeding	Surgical revision
1 (1.6%)	Seroma	Aspiration in outpatient regimen

**Table 3:** Neck metastasis and occult metastasis according to histology.

	Histological Type	pT1-T2 Node Metastasis/Cases	pT3-T4 Node Metastasis/Cases	All Node Metastasis/Cases
**Low risk**	Acinic cell carcinoma	0/13	2/3 (66.6%)	2/16 (12.5%)
	Mucoepidermoid carcinoma (low grade)	0/10	0/2	0/12
	Carcinoma ex pleomorphic adenoma	0/6	1/2 (50%)	1/2 (50%)
	Oncocytic carcinoma	1/1 (100%)	1/2 (50%)	2/3 (66.6%)
	Myoepithelial carcinoma	0/1	0/3	0/4
	Secretory carcinoma	0/3	-	0/3
	**TOTAL LOW RISK**	1/34 (2.9%) *	4/12 (33%) *	5/46 (10.9%) *
**High risk**	Adenocarcinoma	1/1 (100%)	0/1	1/8 (12.5%)
	Mucoepidermoid carcinoma (high grade)	-	0/1	0/1
	Primitive squamous cell carcinoma	1/2 (50%)	0/1	1/3 (33.3%)
	Adenoid cystic carcinoma	0/2	1/6 (16.6%)	1/8 (12.5%)
	Salivary duct carcinoma	1/2 (50%)	-	1/2 (50%)
	High-grade carcinoma NOS ^a^	0/1	1/1 (100%)	1/2 (50%)
	Undifferentiated carcinoma	1/1 (100%)	-	1/1 (100%)
	**TOTAL HIGH RISK**	**4/9 (44.4%)**	**2/10 (20%)**	6/19 (31.6%) *

^a^ NOS: not otherwise specified. * *p* < 0.05.

**Table 4:** Survival rates.

	N	5-Year OS (SE)	5-Year DSS (SE)	5-Year LRFS (SE)	5-Year DMFS (SE)
All patients	64 *	**78.4%** (6.7)	**89%** (4.9)	**92.5%** (3.6)	**87.1%** (4.5)

**OS**: overall survival; **DSS**: disease-specific survival; **LRFS**: local relapse-free survival; **DMFS**: distant metastasis-free survival; **SE**: standard error. * The patient treated for bilateral lesions was evaluated on the basis of the risk factors for the first lesion.

**Table 5:** Univariate analysis of the survival rates according to risk factors.

	N (%)	5-Year DSS (SE)	*p* Value	5-Year LRFS (SE)	*p* Value	5-Year DMFS (SE)	*p* Value
			CI 95%		CI 95%		CI 95%
**Sex** Male	21 (32.8%)	**93.7%** (6.05)	0.6005	**87.8%** (8.06)	0.6817	**89.6%** (6.97)	0.8820
Female	43 (67.2%)	**87.4%** (6.27)	0.08208 to 3.380	**95.2%** (3.32)	0.2480 to 15.72	**86.1%** (5.85)	0.1691 to 3.967
**Age** <55	27 (42.2%)	**89.7%** (7.12)	0.6979	**95.6%** (4.25)	0.3797	**87.2%** (6.94)	0.8222
≥55	37 (57.8%)	**90.4%** (5.30)	0.1392 to 4.715	**89.4%** (5.85)	0.05438 to 2.772	**87.3%** (6.02)	0.2075 to 4.080
**Age** <65	37 (57.8%)	**91.5%** (6.11)	0.3045	**97.3%** (2.66)	0.2313	**89.9%** (5.57)	0.3433
≥65	27 (42.2%)	**87.3%** (6.88)	0.07859 to 2.769	**86.3%** (7.38)	0.03230 to 1.726	**83.6%** (7.57)	0.1194 to 2.410
**Age** <70	47 (73.4%)	**90.6%** (5.45)	0.3591	**93%** (3.90)	0.7700	**90.1%** (4.76)	0.3074
≥70	17 (26.6%)	**86.9%** (8.72)	0.06908 to 3.843	**91.6%** (7.97)	0.1183 to 9.988	**79.4%** (10.73)	0.08367 to 2.501
**Age** <75	54 (84.4%)	**91.8%** (4.80)	0.0681	**93.9%** (3.39)	0.7534	**88.8%** (4.76)	0.1956
≥75	10 (15.6%)	**77.1%** (14.41)	0.02013 to 3.058	**83.3%** (15.21)	0.03079 to 8.227	**78.7%** (13.40)	0.04801 to 3.441
**Histology** Low risk	46 (70.8%)	**92.8%** (5.04)	0.0254	**97.8%** (2.15)	0.0296	**91.6%** (4.67)	0.0229
High risk	19 (29.2%)	**81.4%** (9.86)	0.02859 to 1.668	**77%** (11.89)	0.01058 to 1.044	**76.2%** (10.635)	0.04568 to 1.390
**Neck metastases** No	54 (83.1%)	**95.8%** (2.90)	0.0218	**98.1%** (1.86)	0.0019	**96.3%** (2.59)	0.0003
Yes	11 (16.9%)	**59.6%** (19.81)	0.01163 to 1.387	**64.2%** (16.79)	0.0037 to 0.914	**51.1%** (15.77)	0.01037 to 0.577
**PNI** No	47 (72.3%)	**93.9%** (4.35)	0.0301	**95.2%** (3.31)	0.1328	**92.4%** (4.28)	0.0141
Yes	18 (27.7%)	**76.9%** (12.10)	0.02069 to 1.526	**83.3%** (11.19)	0.02270 to 2.921	**72.4%** (12.208)	0.03418 to 1.251
**LVI** No	58 (89.2%)	**94.7%** (3.85)	<0.0001	**92.3%** (3.71)	0.6232	**93.5%** (3.68)	<0.0001
Yes	7 (10.8%)	**51.4%** (20.38)	0.0021 to 1.486	**100%**	/	**35.7%** (19.79)	0.0027 to 1.043
**Skin involvement** No	61 (93.8%)	**90.1%** (4.76)	0.0545	**92.2%** (3.77)	0.6337	**90.4%** (4.11)	0.0086
Yes	4 (6.2%)	**66.7%** (21.21)	0.0030 to 12.55	**100%**	/	**37.5%** (28.64)	0.00410 to 3.996
**Preoperative facial pain** No	48 (73.8%)	**94.9%** (3.55)	0.0665	**92.5%** (4.16)	0.8153	**90.3%** (4.64)	0.2323
Yes	17 (26.2%)	**76.9%** (12.46)	0.02882 to 1.672	**93.7%** 6.05	0.09260 to 9.327	**79.1%** (11.05)	0.07614 to 2.408
**Facial nerve involvement** No	54 (83.1%)	**92%** (4.56)	0.0683	**91.6%** (4.04)	0.4302	**91.3%** (4.19)	0.0210
Yes	11 (16.9%)	**81.8%** (11.62)	0.02016 to 3.058	**100%**	/	**68.2%** (15.77)	0.02569 to 1.836
**ENE+ in pN+ patients (n = 11)** No	6 (54.5%)	**53.3%** (24.82)	0.7728	**41.7%** (22.17)	0.1360	**62.5%** (21.34)	0.3991
Yes	5 (45.5%)	**80%** (17.88)	0.1064 to 11.88	**100%**	/	**40%** (21.90)	0.08391 to 2.884
**Stage** * I	17 (26.2%)	**100%**	>0.999	**100%**	0.4142	**100%**	>0.999
II	22 (33.8%)	**100%**	/	**94.4%** (5.39)	0.00345 to 10.32	**100%**	/
I	17 (26.2%)	**100%**	>0.999	**100%**	0.0583	**100%**	>0.999
III	10 (15.4%)	**100%**	/	**80%** (12.69)	0.00339 to 1.103	**100%**	/
I	17 (26.2%)	**100%**	0.0137	**100%**	0.0854	**100%**	0.0030
IV	16 (24.6%)	**54.1%** (18.03)	0.01844 to 0.633	**82%** (11.69)	0.00493 to 1.414	**49.9%** (13.75)	0.02344 to 0.464
II	22 (33.8%)	**100%**	>0.999	**94.4%** (5.39)	0.1414	**100%**	>0.999
III	10 (15.4%)	**100%**	/	**80%** (12.69)	0.01230 to 1.872	**100%**	/
II	22 (33.8%)	**100%**	0.0028	**94.4%** (5.39)	0.2038	**100%**	0.0004
IV	16 (24.6%)	**54.1%** (18.03)	0.00957 to 0.380	**82%** (11.69)	0.01836 to 2.346	**49.9%** (13.75)	0.01278 to 0.284
III	10 (15.4%)	**100%**	0.0590	**80%** (12.69)	0.7367	**100%**	0.0260
IV	16 (24.6%)	**54.1%** (18.03)	0.02995 to 1.068	**82%** (11.69)	0.1889 to 10.55	**49.9%** (13.75)	0.03828 to 0.766
**Stage** I+II	39 (59.7%)	**100%**	0.0053	−1.000 to 1.000	0.02	**100%** (4.80)	0.0007
III+IV	26 (40.3%)	**72.1%** (11.95)	−1.000 to 1.000	**81.6%** (8.37)	0.02082to 0.832	**68.4%** (10.14)	−1.000 to 1.000
**Margin** ** R0	43 (66.2%)	**100%**	0.0021	**94.4%** (3.85)	0.0715	**100%**	0.0053
R close	13 (20%)	**75%** (15.30)	8.330 × 10^−5^ to 0.1243	**81.8%** (11.62)	0.00625 to 1.237	**81.4%** (11.93)	0.00024 to 0.234
R0	43 (66.2%)	**100%**	<0.0001	**94.4%** (3.85)	0.5780	**100%**	<0.0001
R1	9 (13.8%)	**38.8%** (28.35)	2.988 × 10^−5^ to 0.02052	**100%**	0.05425 to 185.6	**40%** (17.38)	0.00010 to 0.014
R close	13 (20%)	**75%** (15.30)	0.3600	**81.8%** (11.62)	0.2166	**81.4%** (11.93)	0.1346
R1	9 (13.8%)	**38.8%** (28.35)	0.07117 to 2.612	**100%**	0.3555 to 95.77	**40%** (17.38)	0.07128 to 1.425
**Tumour size** ** ≤2	23 (35.4%)	**95.4%** (4.41)	0.4439	**100%**	0.1766	**95.4%** (4.41)	0.2102
>2 and ≤4	37 (56.9%)	**84.5%** (7.61)	0.07929 to 3.036	**90%** (5.53)	0.01939 to 2.065	**80.8%** (7.13)	0.08102 to 1.739
≤2	23 (35.4%)	**95.4%** (4.41)	0.6336	**100%**	0.0404	**95.4%** (4.41)	0.6336
>4	5 (7.7%)	**100%**	0.02196 to 530.1	**80%** (17.89)	3.818 × 10^−5^ to 0.7971	**100%**	0.02196 to 530.1
>2 and ≤4	37 (56.9%)	**84.5%** (7.61)	0.4436	**90%** (5.53)	0.4265	**80.8%** (7.13)	0.3389
>4	5 (7.7%)	**100%**	0.1673 to 59.34	**80%** (17.89)	0.01453 to 5.984	**100%**	0.2979 to 33.74

**OS**: overall survival; **DSS**: disease-specific survival; **LRFS**: local relapse-free survival; **DMFS**: distant metastasis-free survival; **SE**: standard error; **HR**: hazard ratio; **CI**: confidence interval; **LVI**: lymphovascular invasion; **PNI**: perineural invasion. * *p* value < 0.008 (obtained with Bonferroni’s method) was considered significant. ** *p* value < 0.0167 (obtained with Bonferroni’s method) was considered significant. Significative *p* values are underlined.

## Data Availability

Data is contained within the Supplementary Material.

## Supplementary Materials

The following supporting information can be downloaded at: https://www.mdpi.com/article/10.3390/curroncol30030204/s1, File S1: Multivariate analysis; File S2: Primitive parotid malignancies database.

## References

[1] Carta F., Chuchueva N., Gerosa C., Sionis S., Caria R.A., Puxeddu R. (2017). Parotid tumours: Clinical and oncologic outcomes after microscope-assisted parotidectomy with intraoperative nerve monitoring. Acta Otorhinolaryngol. Ital..

[2] Sung H., Ferlay J., Siegel R.L., Laversanne M., Soerjomataram I., Jemal A., Bray F. (2021). Global cancer statistics 2020: GLOBOCAN estimates of incidence and mortality worldwide for 36 cancers in 185 countries. CA Cancer J. Clin..

[3] Mannelli G., Franchi A., Fasolati M., Cecconi L., Bettiol A., Vannacci A., Gallo O. (2022). Nomograms predictive for oncological outcomes in malignant parotid tumours: Recurrence and mortality rates of 228 patients from a single institution. Eur. Arch. Otorhinolaryngol..

[4] Salivary Gland—Recent Trends in SEER Age-Adjusted Incidence Rates, 2000–2019. https://seer.cancer.gov/statistics-network/explorer/application.html?site=7&data_type=1&graph_type=2&compareBy=sex&chk_sex_1=1&chk_sex_3=3&chk_sex_2=2&hdn_rate_type=1&race=1&age_range=1&stage=101&advopt_precision=1&advopt_show_ci=on&hdn_view=0&advopt_show_apc=on&advopt_display=2#resultsRegion0.

[5] Wang J.R., Bell D., Ferrarotto R., Weber R.S., Su S.Y., Witt R.L. (2019). Malignant Tumors. Surgery of the Salivary Glands.

[6] Zheng R., Wang L., Bondy M.L., Wei Q., Sturgis E.M. (2004). Gamma radiation sensitivity and risk of malignant and benign salivary gland tumors. Cancer.

[7] Horn-Ross P.L., Ljung B.M., Morrow M. (1997). Enviromental factors and risk of salivary gland cancer. Epidemiology.

[8] Gao M., Hao Y., Huang M.X., Ma D.Q., Chen Y., Luo H.Y., Gao Y., Cao Z.Q., Peng X., Yu G.Y. (2017). Salivary gland tumours in a northern Chinese population: A 50-year retrospective study of 7190 cases. Int. J. Oral Maxillofac. Surg..

[9] El-Naggar A.K., Chan J.K.C., Grandis J.R., Takata T., Slootweg P.J. (2017). WHO Classification of Head and Neck Tumours.

[10] Molteni G., Molinari G., Ghirelli M., Sprio A.E., Berta G.N., Malagoli A., Marchioni D., Presutti L. (2019). Oncological outcomes of parotid gland malignancies: A retrospective analysis of 74 patients. J. Stomatol. Oral. Maxillofac. Surg..

[11] Puxeddu I., Capecchi R., Carta F., Tavoni A.G., Migliorini P., Puxeddu R. (2018). Salivary gland pathology in IgG4-related disease: A comprehensive review. J. Immunol. Res..

[12] Dunn E.J., Kent T., Hines J., Cohn I. (1976). Parotid neoplasms: A report of 250 cases and review of the literature. Ann. Surg..

[13] Thielker J., Grosheva M., Ihrler S., Witting A., Guntinas-Lichius O. (2018). Contemporary management of benign and malignant parotid tumors. Front. Surg..

[14] Rossi E.D., Faquin W.C., Baloch Z., Barkan G.A., Foschini M.P., Pusztaszeri M., Vielh P., Kurtycz D.F.I. (2017). The Milan System for reporting salivary gland cytopathology: Analysis and suggestions of initial survey. Cancer Cytopathol..

[15] Amin M., Greene F.L., Edge S.B., Compton C.C., Gershenwald J.E., Brookland R.K., Meyer R., Gress D.M., Byrd D.R., Winchester D.P. (2017). AJCC Cancer Staging Manual.

[16] Quer M., Guntinas-Lichius O., Marchal F., Vander Poorten V., Chevalier D., León X., Eisele D., Dulguerov P. (2016). Classification of parotidectomies: A proposal of the European salivary gland society. Eur. Arch. Otorhinolaryngol..

[17] NCCN Clinical Practice Guidelines in Oncology (NCCN Guidelines®) Head and neck cancers, version 1.2022. https://www.nccn.org/professionals/physician_gls/pdf/head-and-neck.pdf.

[18] House J.W., Brackmann D.E. (1985). Facial nerve grading system. Otolaryngol. Head Neck Surg..

[19] Cho J.K., Lim B.W., Kim E.H., Ko Y.H., Oh D., Noh J.M., Ahn Y.C., Baek K.H., Jeong H.S. (2016). Low-grade salivary gland cancers: Treatment outcomes, extent of surgery and indications for postoperative adjuvant radiation therapy. Ann. Surg. Oncol..

[20] Stodulski D., Mikaszewski B., Majewska H., Wiśniewski P., Stankiewicz C. (2017). Close surgical margin after conservative parotidectomy in early stage low-/intermediate-grade parotid carcinoma: Outcome of watch and wait policy. Oral Oncol..

[21] Seethala R.R. (2009). An update on grading of salivary gland carcinomas. Head Neck Pathol..

[22] Niazi M., Mohammadzadeh M., Aghazadeh K., Sharifian H., Karimi E., Shakiba M., Baniasadi M., Rahmaty B., Adel S., Moharreri M. (2020). Perfusion computed tomography scan imaging in differentiation of benign from malignant parotid lesions. Int. Arch. Otorhinolaryngol..

[23] Liu C.C., Jethwa A.R., Khariwala S.S., Johnson J., Shin J.J. (2016). Sensitivity, specificity, and posttest probability of parotid fine-needle aspiration: A systematic review and meta-analysis. Otolaryngol. Head Neck Surg..

[24] Nakano T., Yasumatsu R., Kogo R., Hashimoto K., Asai K., Ohga S., Yamamoto H., Nakashima T., Nakagawa T. (2019). Parotid gland carcinoma: 32 years’ experience from a single institute. J. Laryngol. Otol..

[25] Sood S., McGurk M., Vaz F. (2016). Management of salivary gland tumours: United Kingdom national multidisciplinary guidelines. J. Laryngol. Otol..

[26] De Vincentis M., Magliulo G., Soldo P., Manciocco V., Pagliuca G., Del Gaizo R., Gallo A. (2005). Extended parotidectomy. Acta Otorhinolaryngol. Ital..

[27] Carta F., Mariani C., Marrosu V., Gerosa C., Puxeddu R. (2020). Three-dimensional, high-definition exoscopic parotidectomy: A valid alternative to magnified-assisted surgery. Br. J. Oral. Maxillofac. Surg..

[28] Guntinas-Lichius O., Silver C.E., Thielker J., Bernal-Sprekelsen M., Bradford C.R., De Bree R., Kowalski L.P., Olsen K.D., Quer M., Rinaldo A. (2018). Management of the facial nerve in parotid cancer: Preservation or resection and reconstruction. Eur. Arch. Otorhinolaryngol..

[29] Park W., Park J., Park S.I., Kim H., Bae H., Cho J., Won H., Park M., Jeon H.S. (2019). Clinical outcomes and management of facial nerve in patients with parotid gland cancer and pretreatment facial weakness. Oral Oncol..

[30] Klussmann J.P., Ponert T., Mueller R.P., Dienes H.P., Guntinas-Lichius O. (2008). Patterns of lymph node spread and its influence on outcome in resectable parotid cancer. Eur. J. Surg. Oncol..

[31] Lombardi D., McGurk M., Vander Poorten V., Guzzo M., Accorona R., Rampinelli V., Nicolai P. (2017). Surgical treatment of salivary malignant tumors. Oral Oncol..

[32] Kaura A., Kennedy R.A., Ali S., Odell E., Simo R., Jeannon J.P., Oakley R. (2019). Utility of neck dissection for management of carcinoma of the parotid gland. Br. J. Oral Maxillofac. Surg..

[33] Westergaard-Nielsen M., Rosenberg T., Gerke O., Dyrvig A.K., Godballe C., Bjørndal K. (2020). Elective neck dissection in patients with salivary gland carcinoma: A systematic review and meta-analysis. J. Oral Pathol. Med..

[34] Koto M., Hasegawa A., Takagi R., Ikawa H., Naganawa K., Mizoe J., Jingu K., Tsujii H., Tsuji H., Kamada T. (2017). Definitive carbon-ion radiotherapy for locally advanced parotid gland carcinomas. Head Neck.

[35] Azami Y., Hayashi Y., Nakamura T., Kimura K., Yamaguchi H., Ono T., Takayama K., Hirose K., Yabuuchi T., Suzuki M. (2019). Proton beam therapy for locally recurrent parotid gland cancer. Indian J. Otolaryngol. Head Neck Surg..

[36] Mueller S.K., Haderlein M., Lettmaier S., Agaimy A., Haller F., Hecht M., Fietkau R., Iro H., Mantsopoulos K. (2022). Targeted Therapy, Chemotherapy, Immunotherapy and Novel Treatment Options for Different Subtypes of Salivary Gland Cancer. J. Clin. Med..

