# Supplementary material for: Survival in Patients with Primary Parotid Gland Carcinoma after Surgery—Results of a Single-Centre Study
Source: Curr Oncol. 2023 Feb 23;30(3):2702–14. doi: 10.3390/curroncol30030204 (PMC10047368; doi:10.3390/curroncol30030204)
Supplement: Supplementary file 1 [file curroncol-30-00204-s001.zip › curroncol-2195003-Supplementary File S1-Multivariate analysis.pdf]

Dependent Y	DSS

Method	Enter

Sample size	64
Positive cases [a]	5 (7,81%)
Negative cases [b]	59 (92,19%)

[a] DSS = yes
[b] DSS = no

Overall Model Fit

Null model -2 Log Likelihood	35,093
Full model -2 Log Likelihood	0,0000000264
Chi-squared	35,093
DF	24
Significance level	P = 0,0670
Cox & Snell R^2	0,4221
Nagelkerke R^2	1,0000

Coefficients and Standard Errors

Variable	Coefficient	Std. Error	Wald	P
Angioinvasione_0_no_1_si_="yes"	7,17463	185075,22541	1,502803892843E-9	1,0000
Cut_off_dimensioni_0_≤2cm_1_2cm_e_≤4_2_4_="large"	-0,90840	203429,65836	1,994E-11	1,0000
Cut_off_dimensioni_0_≤2cm_1_2cm_e_≤4_2_4_="small"	4,34592	80217,58182	2,935109960059E-9	1,0000
Deficit_VII_n.c._preoperatorio_0_no_1_si_="yes"	-7,32697	183704,73600	1,59077448869E-9	1,0000
DMFS="yes"	52,15653	202174,35501	0,00000006655	0,9998
Età_all'intervento_in_anni_	0,13786	2323,98138	3,518932489071E-9	1,0000
Interessamentro_cutaneo_1_si_0_no_="yes"	-19,75204	112628,72998	0,00000003076	0,9999
Lobo_coinvolto_1_superficiale_2_profondo_3_entrambi_="both"	-11,01729	150995,97694	5,323765387154E-9	0,9999
Lobo_coinvolto_1_superficiale_2_profondo_3_entrambi_="deep"	-15,10948	166984,06747	8,187459887966E-9	0,9999

LRFS="yes"	6,33252	1850328842366,55000	0,0000	1,0000
Metastasi_linfonodali_l.c.="yes"	-19,22899	123052,86411	0,00000002442	0,9999
Neuroinvasione_0_no_1_si_="yes"	-1,70316	70876,84995	5,7743E-10	1,0000
OS="yes"	23,10826	102888,34396	0,00000005044	0,9998
R_state_0_R0_1_R_close_1mm_2_R_="close"	1,33937	67364,94247	3,953E-10	1,0000
R_state_0_R0_1_R_close_1mm_2_R_="involved"	10,71130	102545,07269	0,00000001091	0,9999
Recidiva_su_N_0_no_1_si_="yes"	31,65143	1850328842366,56000	0,0000	1,0000
Recidiva_su_T_0_no_1_si_="yes"	-0,14324	1850328842366,55000	0,0000	1,0000
Rischio_istotipo_0_low_risk_1_high_risk_Seethala_2009_="high"	-9,86631	100032,18206	9,728154594647E-9	0,9999
Sacrificio_nervo_faciale_0_no_1_parziale_2_completo_="yes"	-4,65276	67623,17788	4,734012209525E-9	0,9999
Sesso="Male"	-13,84381	51982,25042	0,00000007093	0,9998
Stadio="advanced"	-12,73290	450194609611,75800	0,0000	1,0000
Staging_AJCC_2018="I"	-4,27615	95721,71672	1,995649603405E-9	1,0000
Staging_AJCC_2018="III"	17,93517	450194609611,73900	0,0000	1,0000
Staging_AJCC_2018="IV"	3,56355	450194609611,74700	0,0000	1,0000
Constant	-31,43204	139572,36449	0,00000005072	0,9998

Odds Ratios and 95% Confidence Intervals

Variable	Odds ratio	95% CI
Angioinvasione_0_no_1_si_="yes"	1305,8754	
Cut_off_dimensioni_0_≤2cm_1_2cm_e_≤4_2_4_="large"	0,4032	
Cut_off_dimensioni_0_≤2cm_1_2cm_e_≤4_2_4_="small"	77,1630	
Deficit_VII_n.c._preoperatorio_0_no_1_si_="yes"	0,0007	
DMFS="yes"	44,8E+021	
Età_all'intervento_in_anni_	1,1478	
Interessamentro_cutaneo_1_si_0_no_="yes"	2,6412E-9	

Lobo_coinvolto_1_superficiale_2_profondo_3_entrambi_="both"	0,0000	
Lobo_coinvolto_1_superficiale_2_profondo_3_entrambi_="deep"	0,0000	
LRFS="yes"	562,5732	
Metastasi_linfonodali_l.c.="yes"	4,4561E-9	
Neuroinvasione_0_no_1_si_="yes"	0,1821	
OS="yes"	10,9E+009	
R_state_0_R0_1_R_close_1mm_2_R_="close"	3,8166	
R_state_0_R0_1_R_close_1mm_2_R_="involved"	44859,7729	
Recidiva_su_N_0_no_1_si_="yes"	55,7E+012	
Recidiva_su_T_0_no_1_si_="yes"	0,8665	
Rischio_istotipo_0_low_risk_1_high_risk_Seethala_2009_="high"	0,0001	
Sacrificio_nervo_faciale_0_no_1_parziale_2_completo_="yes"	0,0095	
Sesso="Male"	0,0000	
Stadio="advanced"	0,0000	
Staging_AJCC_2018="I"	0,0139	
Staging_AJCC_2018="III"	61,5E+006	
Staging_AJCC_2018="IV"	35,2883	

Hosmer & Lemeshow test

Chi-squared	2,736046568483E-9
DF	9
Significance level	P = 1,0000

Contingency table for Hosmer & Lemeshow test [Hide]

Group	Y=0		Y=1		Total
	Observed	Expected	Observed	Expected	
1	6	6,000	0	0,000	6
2	6	6,000	0	0,000	6
3	6	6,000	0	0,000	6
4	6	6,000	0	0,000	6
5	6	6,000	0	0,000	6
6	6	6,000	0	0,000	6
7	6	6,000	0	0,000	6
8	6	6,000	0	0,000	6
9	6	6,000	0	0,000	6
10	5	5,000	1	1,000	6
11	0	0,000	4	4,000	4

Classification table (cut-off value p=0,5)

Actual group	Predicted group		Percent correct
	0	1	
Y = 0	59	0	100,00%
Y = 1	0	5	100,00%
Percent of cases correctly classified			100,00%

ROC curve analysis

Area under the ROC curve (AUC)	1,000
Standard Error	0,000
95% Confidence interval	0,944 to 1,000

Dependent Y	DSS
Method	Enter

Sample size	64
Positive cases [a]	5 (7,81%)
Negative cases [b]	59 (92,19%)

[a] DSS = yes
[b] DSS = no

Overall Model Fit

Null model -2 Log Likelihood	35,093
Full model -2 Log Likelihood	0,0000000501
Chi-squared	35,093
DF	19
Significance level	P = 0,0136
Cox & Snell R^2	0,4221
Nagelkerke R^2	1,0000

Coefficients and Standard Errors

Variable	Coefficient	Std. Error	Wald	P
Angioinvasione_0_no_1_si_="yes"	29,84458	98407,24403	0,00000009198	0,9998
Cut_off_dimensioni_0_≤2cm_1_2cm_e_≤4_2_4_="large"	-69,87885	51491,47851	0,000001842	0,9989
Cut_off_dimensioni_0_≤2cm_1_2cm_e_≤4_2_4_="small"	-1,01027	88193,55677	1,3122E-10	1,0000
Deficit_VII_n.c._preoperatorio_0_no_1_si_="yes"	-85,28840	117526,51264	0,0000005266	0,9994
Età_all'intervento_in_anni_	0,081287	1280,56117	4,029426165455E-9	0,9999
Interessamentro_cutaneo_1_si_0_no_="yes"	-42,81504	45137,99593	0,0000008997	0,9992
Lobo_coinvolto_1_superficiale_2_profondo_3_entrambi_="both"	69,87726	130420,27096	0,0000002871	0,9996
Lobo_coinvolto_1_superficiale_2_profondo_3_entrambi_="deep"	-43,58631	41112,40339	0,000001124	0,9992
Metastasi_linfonodali_l.c.="yes"	-85,61998	150610,98458	0,0000003232	0,9995
Neuroinvasione_0_no_1_si_="yes"	-57,25989	90357,39968	0,0000004016	0,9995
R_state_0_R0_1_R_close_1mm_2_R_="close"	-0,88855	82283,45594	1,1661E-10	1,0000
R_state_0_R0_1_R_close_1mm_2_R_="involved"	-26,29552	98631,43763	0,00000007108	0,9998

Rischio_istotipo_0_low_risk_1_high_risk_Seethala_2009_="high"	27,37336	67776,78751	0,0000001631	0,9997
Sacrificio_nervo_faciale_0_no_1_parziale_2_completo_="yes"	-1,38840	37946,94737	1,33867824463E-9	1,0000
Sesso="Male"	-40,32786	62645,73493	0,0000004144	0,9995
Stadio="advanced"	37,55201	450637496693,38900	0,0000	1,0000
Staging_AJCC_2018="I"	1,26581	91933,00325	1,8958E-10	1,0000
Staging_AJCC_2018="III"	-64,50719	450637496693,39000	0,0000	1,0000
Staging_AJCC_2018="IV"	48,90909	450637496693,38300	0,0000	1,0000
Constant	-27,44762	73855,88908	0,0000001381	0,9997

Odds Ratios and 95% Confidence Intervals

Variable	Odds ratio	95% CI
Angioinvasione_0_no_1_si_="yes"	9,15E+012	
Cut_off_dimensioni_0_≤2cm_1_2cm_e_≤4_2_4_="large"	4,4875E-31	
Cut_off_dimensioni_0_≤2cm_1_2cm_e_≤4_2_4_="small"	0,3641	
Deficit_VII_n.c._preoperatorio_0_no_1_si_="yes"	9,1142E-38	
Età_all'intervento_in_anni_	1,0847	
Interessamentro_cutaneo_1_si_0_no_="yes"	2,5449E-19	
Lobo_coinvolto_1_superficiale_2_profondo_3_entrambi_="both"	2,2249E+30	
Lobo_coinvolto_1_superficiale_2_profondo_3_entrambi_="deep"	1,1768E-19	
Metastasi_linfonodali_l.c.="yes"	6,5421E-38	
Neuroinvasione_0_no_1_si_="yes"	1,3563E-25	
R_state_0_R0_1_R_close_1mm_2_R_="close"	0,4113	
R_state_0_R0_1_R_close_1mm_2_R_="involved"	3,8019E-12	
Rischio_istotipo_0_low_risk_1_high_risk_Seethala_2009_="high"	773E+009	
Sacrificio_nervo_faciale_0_no_1_parziale_2_completo_="yes"	0,2495	
Sesso="Male"	3,0608E-18	
Stadio="advanced"	20,4E+015	
Staging_AJCC_2018="I"	3,5460	
Staging_AJCC_2018="III"	9,658E-29	
Staging_AJCC_2018="IV"	1,7416E+21	

Hosmer & Lemeshow test

Chi-squared	5,157705291009E-9
DF	9
Significance level	P = 1,0000

Contingency table for Hosmer & Lemeshow test [Hide]

Group	Y=0		Y=1		Total
	Observed	Expected	Observed	Expected	
1	6	6,000	0	0,000	6
2	6	6,000	0	0,000	6

3	6	6,000	0	0,000	6
4	6	6,000	0	0,000	6
5	6	6,000	0	0,000	6
6	6	6,000	0	0,000	6
7	6	6,000	0	0,000	6
8	6	6,000	0	0,000	6
9	6	6,000	0	0,000	6
10	5	5,000	1	1,000	6
11	0	0,000	4	4,000	4

Classification table (cut-off value p=0,5)

Actual group	Predicted group		Percent correct
	0	1	
Y = 0	59	0	100,00%
Y = 1	0	5	100,00%
Percent of cases correctly classified			100,00%

ROC curve analysis

Area under the ROC curve (AUC)	1,000
Standard Error	0,000
95% Confidence interval	0,944 to 1,000
